# Effect of Acceptor Traps in GaN Buffer Layer on Breakdown Performance of AlGaN/GaN HEMTs

**DOI:** 10.3390/mi14010079

**Published:** 2022-12-28

**Authors:** Maodan Ma, Yanrong Cao, Hanghang Lv, Zhiheng Wang, Xinxiang Zhang, Chuan Chen, Linshan Wu, Ling Lv, Xuefeng Zheng, Wenchao Tian, Xiaohua Ma, Yue Hao

**Affiliations:** 1 School of Electronics & Mechanical Engineering, Xidian University, Xi’an 710071, China; 2 State Key Discipline Laboratory of Wide Bandgap Semiconductor Technology, School of Microelectronics, Xidian University, Xi’an 710071, China

**Keywords:** AlGaN/GaN HEMTs, acceptor traps, Silvaco TCAD, breakdown voltage, leakage current, additional electric field

## Abstract

In this paper, Silvaco TCAD software is used to simulate the buffer traps in AlGaN/GaN high electron mobility transistors (HEMTs), and its effects on the breakdown performance and key parameters of the devices are investigated by changing the position and concentration of the acceptor traps in the buffer layer. The results show that with the increase of trap concentration, the traps capture electrons and reduce the off-state leakage current, which can improve breakdown voltage of the devices. At the same time, as the trap concentration increases, the ionized traps make a high additional electric field near the drain edge, leading to the decrease of breakdown voltage. With the combined two effects above, the breakdown voltage almost ultimately saturates. When the source-to-gate (Access-S) region in the GaN buffer layer is doped alone, the minimum and most linear leakage current for the same trap concentrations are obtained, and the additional electric field has a relatively small effect on the electric field peak near the drain as the ionized traps are furthest from drain. All these factors make the breakdown voltage increase more controllably with the Access-S region doping, and it is a more potential way to improve the breakdown performance.

## 1. Introduction

The AlGaN/GaN high electron mobility transistors (HEMTs) are considered to be promising candidates for the next generation of high power and high frequency devices, due to their outstanding combination of fundamental physical properties, such as large breakdown fields, high two-dimensional electron gas (2DEG) density, low on-state resistance, and high electron mobility [1,2]. In recent years, GaN power electronic devices have received wide attention in microwave and power applications [3,4]. However, under the standard growth conditions, n-type background carriers are introduced within the GaN buffer layer, leading to an increase of leakage current, which in turn leads to poor breakdown performance of the devices. Breakdown voltage of the devices determines the maximum operating voltage and output power [5]. Therefore, in order to achieve a semi-insulating GaN buffer layer, compensation doping in the GaN buffer layer is a common technique. Through density functional theory-based calculations, it is shown that C (or Fe) has a higher probability to replace N due to lower formation energy required for C-N (or C-Fe) substitution in the GaN buffer layer [6]. As a result, the injection of impurities (Fe, C, etc.) into the GaN buffer layer are often used in the manufacturing process to form acceptor traps with deep energy levels [7]. Therefore, the acceptor traps can capture the background electrons to achieve the high resistance characteristic of the buffer layer and reduce the undesirable effect of buffer leakage currents [8]. The effects of the doping concentration in the buffer layer on the DC as well as frequency characteristics of the devices have been studied extensively [8,9]. However, the trend and mechanism of the doping concentration effect on delaying the avalanche breakdown and increasing the breakdown voltage of the devices are not clear. Bahat-Treidel et al. propose that the subthreshold buffer leakage currents are reduced and thus, postpone *V_bd_* (breakdown voltage) to higher voltages by doping in the buffer layer [10]. Zhu et al. believe that with gate-to-drain spacing increasing, the negatively charged buffer traps region spreads wider and the depletion region length becomes longer, playing a key role in the linear dependence of off-state breakdown voltage on gate to drain spacing [11]. Joshi et al. propose that there is an optimum moderate buffer acceptor trap concentration for maximizing the breakdown voltage as a function of gate–drain distance and field plate length [12], but the mechanism of acceptor trap action in the buffer layer is not described. Therefore, it is important to study the influence of the acceptor trap concentration on the breakdown voltage of the AlGaN/GaN HEMTs. Also, selecting a reasonable doping concentration for the reduction of the leakage current and the improvement of breakdown voltage is very beneficial to improve the device performances. In this paper, we investigated the effects of the acceptor trap concentration in the GaN buffer layer on the leakage current and breakdown performance of conventional depletion-mode (D-mode) AlGaN/GaN HEMTs by using Silvaco TCAD software [13]. Furthermore, the physical mechanism and degradation trend were analyzed by combining the device electric field and electrons concentration distribution with simulation plots; the key region affecting the breakdown performance of AlGaN/GaN HEMTs was proposed to provide a theoretical basis for optimizing the breakdown performance of the devices.

## 2. Computational Framework

**Figure 1:**
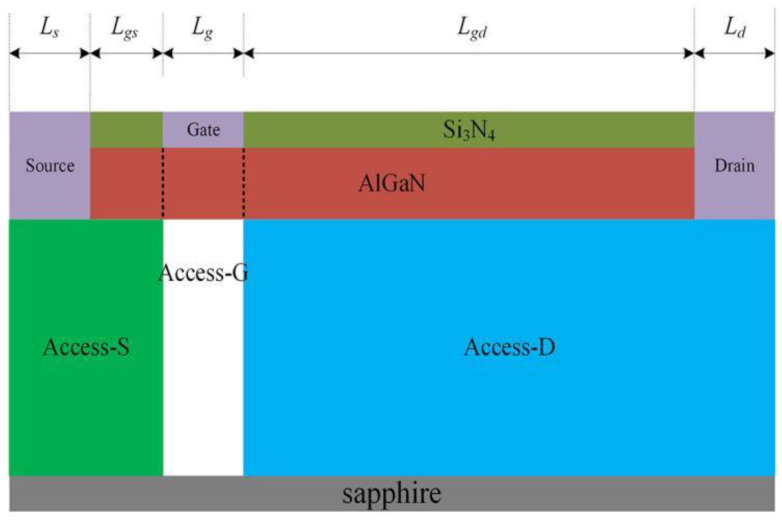
Simulation structure and buffer layer area division.

Based on the reference [14] and the actual manufacturing process, device cross section as shown in Figure 1 is used for computations in this paper. The materials from bottom to top are sapphire substrate, 1 μm GaN buffer layer, 20 nm AlGaN barrier layer with Al component of 0.3, and Si_3_N_4_ passivation material with 60 nm thickness, respectively. In addition, the gate width of the device is 50 μm. A background carrier concentration of 1 × 10^15^ cm^−3^ is added to the GaN buffer layer. The device has Ohmic contacts at the drain and source electrodes. The gate electrode is a Schottky contact; a barrier height of 1.6 eV is considered [15].

The distance (*L_gs_*) of source to gate and the distance (*L_gd_*) of gate to drain are 1 μm and 4 μm, respectively, and the length of gate (*L_g_*), drain (*L_d_*), and source (*L_s_*) electrodes are all 1 μm. The one-dimensional Schrodinger equation is applied to obtain two-dimensional electron gas (2DEG) in the AlGaN/GaN heterostructure [16]. The Shockley–Read–Hall (SRH) recombination model is used to simulate the charging and discharging effect of the acceptor traps. To simulate carrier mobility changes in practice, the doping-dependent mobility model and high-field saturation model are used to calculate the electron mobility, including the low-field mobility model (Albrct.n) and high-field mobility model (Gansat) [17,18]. A fixed temperature of T = 300 K is assumed in simulation because a self-heating effect could be neglected due to off-state working conditions of devices. Furthermore, the Selberherr’s impact ionization model is used in the paper to simulate the breakdown of the buffer layer caused by impact ionization. The material parameters of AlGaN, such as work function, bandgap, and dielectric coefficient, are obtained by linear interpolation between GaN and AlN [6]. In addition, we define the source-to-gate region in the buffer layer as the Access-S (green area in Figure 1), the region below the gate as the Access-G (white area in Figure 1), and the gate-to-drain region in the buffer layer as the Access-D region (blue area in Figure 1). To ensure consistency with the actual manufacturing process, the source and drain electrodes are all etched into the GaN buffer layer in simulation to be able to directly contact the heterojunction channel [19]. Meanwhile, the parameters of the GaN material are set in simulation as shown in Table 1 [20,21].

**Figure 2:**
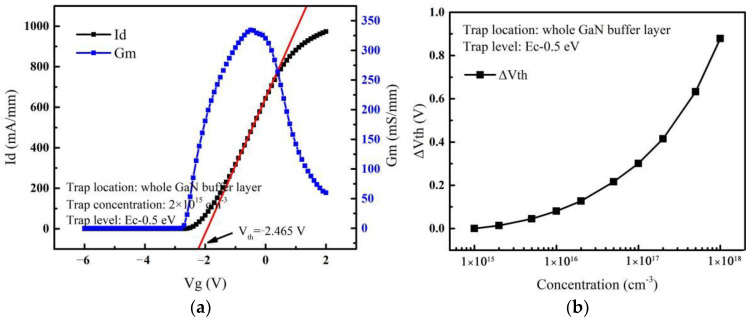
(**a**) Transfer and transconductance curves. (**b**) The variation of threshold voltage with acceptor trap concentrations.

When the device is in the off-state, there are two general methods for determining breakdown voltage (*V_bd_*): one is defined as the drain voltage corresponding to a drain current of 1 mA/mm [22]; the other is defined as the drain voltage corresponding to a sharp rise in the drain current [23], which is also the way to determine the occurrence of breakdown in this paper. To ensure that the trap parameters in the buffer layer are consistent with the trap introduced by the actual doping, only the acceptor traps are added to the buffer layer. The trap energy level and capture cross section are E_c_ −0.5 eV and 1 × 10^−15^ cm^−2^, respectively [24,25]. As the GaN buffer layer doping process introduces the acceptor trap concentration in the range of 10^15^ cm^−3^ to 10^18^ cm^−3^ in the actual process [26,27], we define that the concentration of the acceptor traps varies from 1 × 10^15^ cm^−3^ to 1 × 10^18^ cm^−3^ in each region. The transfer and transconductance curves of the device are shown in Figure 2a. Transductance is the differential of the drain current (*I_d_*) to the gate voltage (*V_g_*), reflecting the ability of the gate electrode to control the drain current. The threshold voltage obtained from the transfer curve is −2.465 V and the maximum transconductance is 334.3 mS/mm, which meet the simulation requirements for depletion-mode devices [7,26]. The variation of threshold voltage with acceptor trap concentrations is shown in Figure 2b; it can be seen that the threshold voltage increases with the increase of trap concentration, which is consistent with others’ research [9,12,27]. Therefore, the structure can be used as the simulation of breakdown performance.

The off-state gate voltage is set to –6 V in the simulation of breakdown performance. When the device is in the off-state, some unexpected electrons will still flow to the drain electrode, and the drain current will increase with the increase of drain voltage. When the device breaks down, the drain current will rise sharply. Therefore, when the device is in the off-state, we call the leakage drain current in the breakdown curve as the off-state leakage current. We define *I_d-leak_* (the parameter of leakage current) as the drain current, corresponding to *V_d_* = *V_bd_*/2 in the breakdown curve [27], and the expression is shown below.

(1) $$I_{d-leak}=I_{d}@(V_{d}=V_{bd}/2)$$

## 3. Effect of Traps in the Whole Buffer Layer

**Figure 3:**
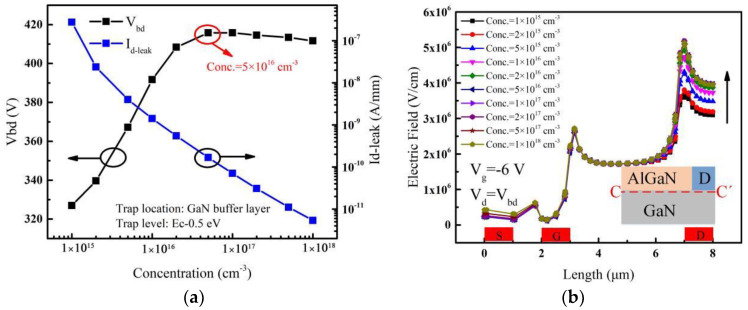
(**a**) Effect of acceptor trap concentration in the whole buffer layer on *V_bd_* and *I_d-leak_*. (**b**) Electric fields extracted along the lateral line (C-C’) at the onset of avalanche breakdown for different acceptor trap concentrations. Inset: cut-line along which electric fields are extracted.

The breakdown voltage and leakage current of the device with the acceptor traps introduced in the whole buffer layer are shown in Figure 3a. It can be observed that when the acceptor trap energy level is fixed, the breakdown voltage (*V_bd_*) increases and gradually saturates with the increase of trap concentration. Its maximum value exists around 5 × 10^16^ cm^−3^ and then starts to decrease slightly with the trap concentration increasing. Figure 3b shows the electric field distribution at 1 nm below the heterojunction channel when the device breaks down. As the concentration of acceptor traps in the GaN buffer layer increases, the electric field near the gate is almost unchanged, while it is more variable near the drain edge. This means that the change in the concentration of the traps mainly affects the electric field peak near the drain edge, which in turn affects the variation of breakdown voltage. The breakdown location of AlGaN/GaN HEMTs tends to occur at the electric field peak [28].

It can be seen from Figure 3a that the leakage current (*I_d-leak_*) decreases continuously with the increase of trap concentration. At the same time, the breakdown voltage continues to increase until the acceptor trap concentration reaches 5 × 10^16^ cm^−3^. Under the condition of considering that only electrons capture effect, the SRH capture rate ($R_{\mathrm{net}}^{\mathrm{SRH}}$) can be expressed as [29,30]:

(2) $$R_{\mathrm{net}}^{\mathrm{SRH}}=\frac{p}{\tau_{p}\left[1+\frac{n_{1}}{N_{c}\exp\left(\eta_{n}\right)}\right]}$$

(3) $$n_{1}=n_{i\;}\exp\left(\frac{E_{\mathrm{trap}\;}}{kT}\right);\;\eta_{n}=\frac{E_{c}-E_{F_{n}}}{kT};\;\eta_{n}=\frac{E_{c}-E_{F_{n}}}{kT}$$

where *p* is the concentration of acceptor traps, *τ_p_* is the lifetime of hole, *N_C_* is the effective density of states for electrons, *n_i_* is the intrinsic electron concentration, *E_trap_* is the difference between the trap energy level and the intrinsic Fermi level, *E_Fn_* is the electron quasi-Fermi energy, *E_c_* is the conduction band energy, *k* is Boltzman’s constant, *T* is the lattice temperature. 

**Figure 4:**
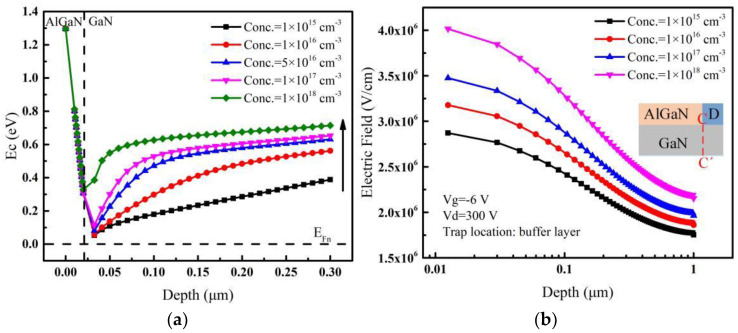
For different acceptor trap concentrations, (**a**) conduction band energy extracted along a vertical line (at the center of the gate electrode) traversing from AlGaN surface (0 nm) to GaN buffer layer. (**b**) Electric fields extracted along the vertical line (C-C’) near the drain edge when the drain voltage is 300 V. Inset: cut-line along which electric fields are extracted.

From Equations (2) and (3), it can be seen that when the acceptor trap energy is fixed (i.e., *E_trap_* is fixed), the electrons’ capture rate increases with the increase of acceptor trap concentration *p* and parameter *η_n_*, and the parameter *η_n_* is proportional to the difference between the conduction band energy (*E_c_*) and the Fermi energy (*E_Fn_*). Figure 4a shows the conduction band energy of the device at different trap concentrations; it can be seen that the Fermi energy (*E_Fn_*) is closer to the conduction band (*E_c_*) when the acceptor trap concentration is lower. That is, *η_n_* is smaller when the trap concentration is lower. In other words, the number of electrons captured by acceptor traps will increase with the high trap concentration, which in turn leads to a decrease in the leakage current in the buffer layer. With the decrease of the leakage current, the resistivity of the buffer layer gradually increases, thus delaying its avalanche breakdown [9,31]. Therefore, the breakdown voltage continues to increase until the trap concentration reaches 5 × 10^16^ cm^−3^.

As the acceptor trap concentration increases, the leakage current continues to decrease. However, when the acceptor trap concentration exceeds 5 × 10^16^ cm^−3^, the breakdown voltage no longer continues to increase as the leakage current decreases, but reaches saturation and even decreases slightly as shown in Figure 3a. This is mainly due to the formation of negative charges (ionized traps) after trapping electrons [32]. The trapping electrons can create an additional built-in electric field whose direction is the same to that applied by the drain voltage [27], and then the whole electric field is strengthened. Therefore, when the drain voltage is fixed, the difference of electric fields with different acceptor trap concentrations in the buffer layer is caused by the additional built-in electric field. As the acceptor traps mainly affect the peak of the electric field near the drain edge (see Figure 3b), the electric fields near the drain edge are extracted along the vertical line (C-C’) and shown in Figure 4b when the drain voltage is fixed at a high value. It can be seen that the electric field near the drain becomes larger with the increase of acceptor trap concentration by the effect of the additional electric field, and the higher the electric field is, the easier the breakdown takes place [28]. Therefore, the additional electric field whose direction is the same to that applied by the drain voltage can make the breakdown voltage decrease.

As discussed above, there are two factors affecting the breakdown voltage. One is that the capture of electrons by the acceptor traps reduces the leakage current and then increases the breakdown voltage of the device. Second is that the ionized traps can generate an additional electric field near the drain edge and the direction is the same to that applied by the drain voltage, resulting in a decrease in the breakdown voltage. Under the combined two effects above, the breakdown voltage almost saturates when the trap concentration increases to 5 × 10^16^ cm^−3^ and then decreases slightly as the additional electric field effect becomes a little larger with the trap concentration continuously increases as shown in the Figure 3a.

To further investigate the availability of region doping in the GaN buffer layer, we will analyze the effects of acceptor traps in the Access-D, Access-G, and Access-S regions on the breakdown performance of the device in the following subsections, respectively.

### 3.1. Effect of Traps in the Access-D Region

**Figure 5:**
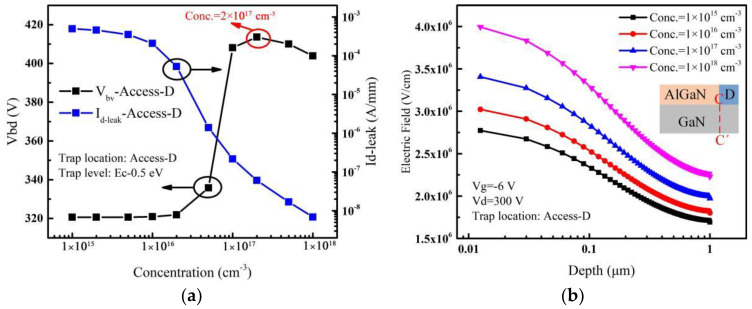
(**a**) Impact of acceptor trap concentration considered in the Access-D region on *V_bd_* and *I_d-leak_*. (**b**) For different acceptor trap concentrations, electric fields extracted along the vertical line (C-C’) near the drain edge when the drain voltage is 300 V. Inset: cut-line along which electric fields are extracted.

When the acceptor traps are only introduced in the Access-D region, the variation of leakage current and breakdown voltage with the acceptor trap concentration are shown in Figure 5a. The breakdown voltage reaches a maximum value by increasing the concentration of traps up to 2 × 10^17^ cm^−3^ and then decreases. The leakage current decreases by about 5 orders of magnitude. For different acceptor trap concentrations, electric fields extracted along the vertical line (C-C’) near the drain edge with a high drain voltage are shown in Figure 5b. It shows that the electric field near the drain increases continuously with the increase of the acceptor trap concentration. As a result, when the trap concentration is lower (before 2 × 10^16^ cm^−3^), a slight decrease in leakage current occurs, resulting in a little change in breakdown voltage. As the trap concentration continues to increase, the leakage current decreases rapidly, causing a rapid increase in the breakdown voltage. Also, because of the higher acceptor trap concentration, a higher additional electric field is introduced near the drain edge [33]. As the acceptor traps in the Access-D region are closer to the drain electrode, the additional electric field formed by ionized traps has a more obvious effect on the electric field peak near the drain. As discussed above, the breakdown voltage starts to drop after saturation as shown in Figure 5a. 

### 3.2. Effect of Traps in the Access-G Region

**Figure 6:**
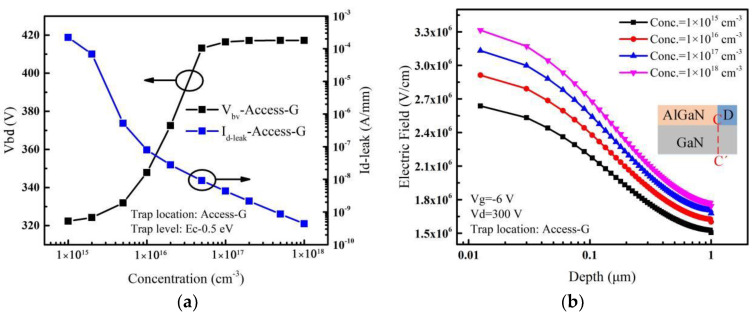
(**a**) Impact of acceptor trap concentration considered in the Access-G region on *V_bd_* and *I_d-leak_*. (**b**) For different acceptor trap concentrations, electric fields extracted along the vertical line (C-C’) near the drain edge when the drain voltage is 300 V. Inset: cut-line along which electric fields are extracted.

When the acceptor traps are only introduced in the Access-G region, the variation of leakage current and breakdown voltage with the acceptor trap concentration are shown in Figure 6a. It can be seen that the breakdown voltage increases first and then gradually saturates as the trap concentration in the Access-G region increases. The leakage current decreases by about 6 orders of magnitude. When the trap concentration is lower, the leakage current decreases rapidly with the acceptor trap concentration increasing, leading to a rapid increase in the breakdown voltage. For different acceptor trap concentrations, electric fields extracted along the vertical line (C-C’) near the drain edge with a high drain voltage are shown in Figure 6b. It can be seen that with the change of the acceptor trap concentration in the Access-G region, the electric field varies less than that with introducing acceptor traps in the Access-D region. This behavior can be explained as follows.

**Figure 7:**
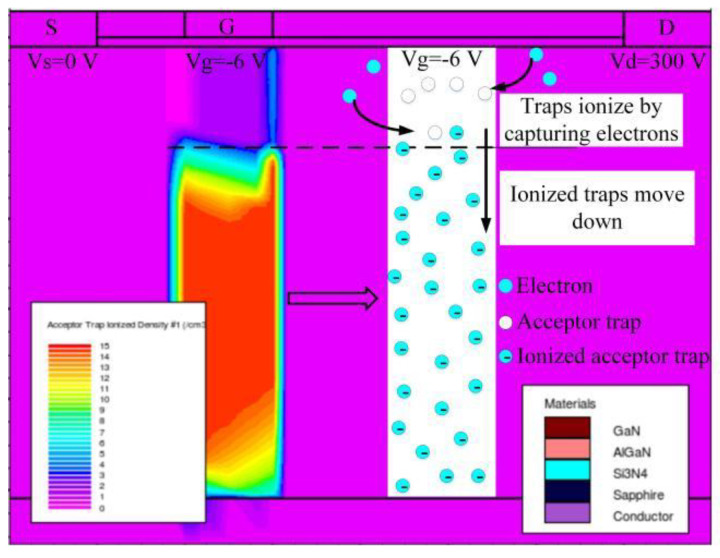
Ionized acceptor traps distribution in the Access-G region.

The distribution of the ionized acceptor traps in the Access-G region is simulated and shown in Figure 7. Ionized traps move down because of the repelling effect of negative gate voltage [32]. Therefore, compared with traps in the Access-D region, the ionized traps in the Access-G region are mainly distributed in the region below the gate and further away from the drain. This results in a relatively small increase of the additional electric field near the drain. Under the combined effect of the decreasing leakage current and the smaller increasing additional electric field near the drain, the breakdown voltage saturates at last (see Figure 6a).

### 3.3. Effect of Traps in the Access-S Region

**Figure 8:**
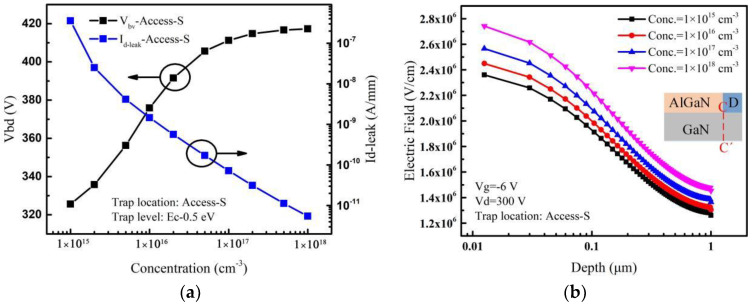
(**a**) Effect of acceptor trap concentration in the Access-S region on *V_bd_* and *I_d-leak_*. (**b**) For different acceptor trap concentrations, electric fields extracted along the vertical line (C-C’) near the drain edge when the drain voltage is 300 V. Inset: cut-line along which electric fields are extracted.

When the acceptor traps are only introduced in the Access-S region, the variation of leakage current and breakdown voltage with the acceptor trap concentration are shown in Figure 8a. The breakdown voltage increases rapidly and then slowly with the increase of the acceptor trap concentration in the Access-S region. The leakage current decreases by about 6 orders of magnitude. With the increase of trap concentration in the Access-S region, the leakage current decreases and the breakdown voltage increases. Meanwhile, ionized traps create an additional electric field in the GaN layer. The electric fields extracted along the lateral line (C-C’) near the drain edge are shown in Figure 8b. The result shows that the electric field near the drain edge varies less with trap concentration compared with that in the Access-D and Access-G regions. This means that the additional electric field generated by the ionized traps has the smallest effect on the electric field peak near the drain edge, as the traps in the Access-S region are farthest from the drain electrode. Therefore, with the smallest effect of the additional electric field, the breakdown voltage still increases slowly instead of dropping or saturation at the larger trap concentrations as shown in Figure 8a.

### 3.4. Comparison of Leakage Current When Traps Are Introduced in Different Regions

The simulation results show that the artificial doping concentrations of Fe or C should be strictly controlled to improve the breakdown voltage. In particular, it is important to avoid the deterioration of the breakdown performance due to the high doping concentration [34,35,36].

**Figure 9:**
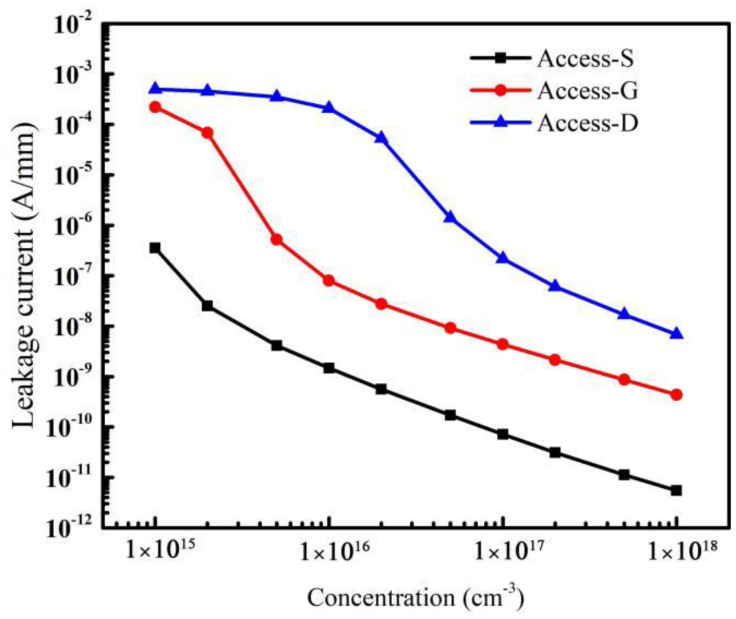
Variations of leakage current caused by traps in different regions.

**Figure 10:**
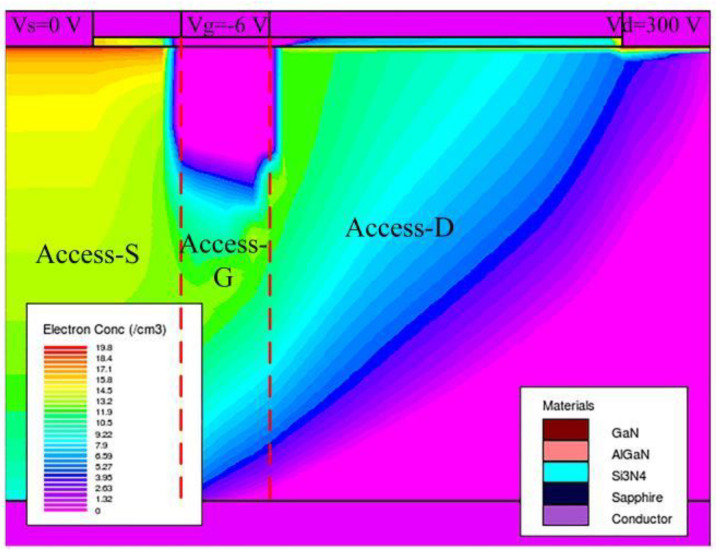
The distribution of electrons inside the device under the off-state voltage.

Figure 9 shows the variations of off-state leakage currents when acceptor traps are introduced in different regions. It can be seen that the traps in the Access-S region cause the minimum leakage current for the same trap concentration. The distribution of electrons inside the device under the off-state gate voltage condition is shown in Figure 10. The flow direction of electrons in the buffer layer is from the source to the drain when the device is at a negative gate voltage and positive drain voltage [37]. Therefore, the electron concentration in the Access-S region is the highest compared with those in the Access-D and Access-G regions as shown in Figure 10, which makes it easier for the acceptor traps in the Access-S region to capture electrons. As a result, for the same trap concentration, the leakage current is relatively small as more electrons are trapped when the traps are located in the Access-S region.

In addition, the variation of the leakage current is directly related to the breakdown voltage of the device [38]. The acceptor traps in the Access-S region can obtain a more uniform variation of the leakage current, and the decreasing trend of the leakage current is almost linear (see Figure 9). Thus, the breakdown voltage rises more gently and evenly with doping in the Access-S region. Moreover, because the acceptor traps in the Access-S region are farthest away from the drain, the additional electric field caused by ionized traps has the smallest effect on the electric field peak near the drain. All factors above make the breakdown voltage increase more controllably. Therefore, it is a potential approach to control breakdown voltage by doping in the Access-S region during the fabrication process, though more research and practice are needed before its practical application.

## 4. Conclusions

This paper focuses on the effect of the concentration and distribution of the acceptor traps in the buffer layer on breakdown performance of the AlGaN/GaN HEMTs. The results show that the acceptor traps can capture electrons generated by ionization collisions and then decrease off-state leakage currents, which result in an increase of the breakdown voltage. At the same time, with the increase of trap concentration, the electric field near the drain will increase as the ionized traps can create an additional built-in electric field whose direction is the same to that applied by the drain voltage. Under the combination of the above two effects, the breakdown voltage almost ultimately saturates even though the leakage current continues to decrease when the traps are introduced in the whole buffer layer. In addition, the effects of traps in the source-to-gate region (Access-S), the region below the gate (Access-G), and the gate-to-drain region (Access-D) on the breakdown performance of the device are studied separately. The results show that the acceptor traps in the Access-S region can cause the minimum and linear leakage current, and the breakdown voltage can be increased more controllably. Therefore, doping in the Access-S region during the fabrication process is a more potential way to improve the breakdown performance of the device than doping in the other two regions.

## Figures and Tables

**Table 1:** The parameters of GaN material in simulation.

Parameters of GaN Material	GaN
the band-gap energy	3.4/eV
the relative dielectric permittivity	9.5
lattice temperature	300/K
electron low-field mobility in GaN layer	900/(cm^2^/(V × s))
saturated velocity of electrons	2 × 10^7^ /(cm/s)
